# Edges in Agricultural Landscapes: Species Interactions and Movement of Natural Enemies

**DOI:** 10.1371/journal.pone.0059659

**Published:** 2013-03-26

**Authors:** Sarina Macfadyen, Warren Muller

**Affiliations:** 1 CSIRO Ecosystem Sciences and the Sustainable Agriculture Flagship, Canberra, Australian Capital Territory, Australia; 2 CSIRO Mathematics, Informatics and Statistics, Canberra, Australian Capital Territory, Australia; Centro de Investigación y de Estudios Avanzados, Mexico

## Abstract

Agricultural landscapes can be characterized as a mosaic of habitat patches interspersed with hostile matrix, or as a gradient of patches ranging from suitable to unsuitable for different species. Arthropods moving through these landscapes encounter a range of edges, with different permeability. Patches of native vegetation in these landscapes may support natural enemies of crop pests by providing alternate hosts for parasitic wasps and/or acting as a source for predatory insects. We test this by quantifying species interactions and measuring movement across different edge-types. A high diversity of parasitoid species used hosts in the native vegetation patches, however we recorded few instances of the same parasitoid species using hosts in both the native vegetation and the crop (canola). However, we did find overall greater densities of parasitoids moving from native vegetation into the crop. Of the parasitoid groups examined, parasitoids of aphids (Braconidae: Aphidiinae) frequently moved from native vegetation into canola. In contrast, parasitoids of caterpillars (Braconidae: Microgastrinae) moved commonly from cereal fields into canola. Late season samples showed both aphids and parasitoids moving frequently out of native vegetation, in contrast predators moved less commonly from native vegetation (across the whole season). The season-long net advantage or disadvantage of native vegetation for pest control services is therefore difficult to evaluate. It appears that the different edge-types alter movement patterns of natural enemies more so than herbivorous pest species, and this may impact pest control services.

## Introduction

Agricultural landscapes are readily conceptualized as a mosaic of suitable habitat patches interspersed with hostile matrix [1], [2]. For many species this simplified version of the landscape is probably similar to what they experience, and the connectivity of habitat patches is crucial to their survival. However, for other species the landscape is more of a gradient of patches that span the full spectrum of suitable to unsuitable. They can utilise resources from both crop and non-crop patches [3], and the decision to move from one place to another is made depending on the risks associated with a particular matrix-type. For example, Cosentino *et al.* [4] showed that desiccation risk to salamanders varied between crops, and the salamanders were more likely to move towards soybean than corn, likely due to reduced water loss under the soybean canopy. For mammals, matrix tolerance is an important predictor of species success in modified landscapes, and increased modification of the matrix can reduce the ability of animals to move through the matrix [5]. The decision to enter into the matrix coincides with the decision to leave the patch and so studying the characteristics of the habitat patch or matrix in isolation may provide only picture partial understanding [6]. Here we have focussed on assessing movement patterns across different landscape boundaries or edges as a means of understanding the degree of suitability of both patch and matrix habitats. Movement across particular edges is influenced by their shape (perimeter-to-area ratios) and their contrast (the degree to which habitat types on each side of the edge differ from one another) [7], [8]. It is hypothesized that edges with a high degree of contrast (“hard” edges) are relatively impermeable to movement, and those with a low degree of contrast (“soft” edges) are more permeable [9]. An empirical study by Collinge and Palmer [7] using ground-dwelling beetles supports this hypothesis, with low contrast boundaries exhibiting net immigration.

Agricultural landscapes are ideal for studying edge movement because most boundaries are abrupt and arbitrarily defined by the farmer. Species encounter a range of edges from high to low contrast, and the degree of contrast changes across the season as the crops develop. Edge-effects have mainly been examined as part of habitat fragmentation studies in agricultural landscapes [6]. In these studies, the process of fragmentation coincides with an overall loss of habitat, and the agricultural matrix is generally a hostile environment [10]. However, many species in these ecosystems are well adapted to using ephemeral and spatially patchy resources and use a variety of habitats throughout their life [11], [12]. For natural enemies of pests, such as predators and parasitic wasps (parasitoids), non-crop vegetation such as windbreaks, field margins, hedgerows and remnant native vegetation may provide alternate hosts or prey species [13]. Corbett and Rosenheim [14] found that an important parasitoid used an alternate host in prune trees planted adjacent to vineyards. Up to 34% of colonizing parasitoids originated from this refuge. However, the positive role of non-crop patches in providing alternate hosts for parasitoids is not always supported by studies examining landscape complexity and species interactions [15], [16].

For natural enemies of pests, we would ideally like edges to be permeable so they can immigrate easily into crop fields when their services are required [11]. Once in the crop, we would like them to attack and kill pest species. Identification and quantification of feeding interactions between pests and their natural enemies provides crucial evidence that individuals moving into a crop field may be providing pest control services [17]. Here we assess species interactions and movement in an agricultural landscape to address two questions. Firstly, do native vegetation patches provide alternative non-pest hosts for important parasitoids? Secondly, do natural enemies move from native vegetation patches into adjacent crop fields? The provision of ecosystem services from native vegetation patches, such as pest control, can be seen as a way of offsetting the opportunity cost associated with retaining native vegetation. Therefore we require a greater movement of natural enemies (and/or less movement of pests) than we would observe if the native vegetation patch was replaced with a crop.

## Materials and Methods

### Ethics Statement

No specific permits were required for the described field studies. Land-holders granted us permission to access their property and conduct the arthropod sampling.

### Hebivore-parasitoid Interactions

We collected and reared insect herbivores (Lepidopteran larvae) from a range of crop and non-crop habitat-patches to construct a quantitative food web. In 2009 we sampled fields and native perennial vegetation (NPV) patches within a circular area of 10 km diameter near Cootamundra NSW (lat. 34.72°S, long. 147.73°E). Transects (1 m width) were laid down monthly in arable fields (wheat, barley and canola), pasture fields and NPV. In total 502 m of cereal transect was searched, 440 m of canola, 600 m of fallow arable field, 260 m of pasture, and 360 m of NPV. In 2010 additional sampling was conducted in a second landscape 76 km to the North-East near Young (lat.34.42°S, long. 148.46°E). At both sites 6 canola fields, 6 wheat fields, 12 pasture fields and 9–12 NPV patches were sampled on each monthly visit. The vegetation was sampled by beating into a box (37 cm×30 cm×12 cm) or using a suction sampler (converted leaf blower Stihl^®^ Sh863, 11 cm diameter pipe placed over the extraction fan). One suction sample consisted of a 120 cm×30 cm area. A sock was placed over the intake tube to collect the insects and these were immediately emptied into a box and examined in the field. In total 720 boxes were sampled in wheat fields, 630 boxes in canola, 2650 suction samples in pasture, and 2797 boxes and suction samples in NPV patches. Herbivores were placed in individual 30 oz cups (with their host plant) and transported back to the laboratory in a chilled cooler-box. Extra plant material was added as the larvae matured in a temperature-controlled glasshouse. Each individual was maintained until it died, an adult host emerged, or a parasitoid emerged. The adult host or parasitoid was pin- (or card-) mounted and sorted into families or morphospecies and further identified by taxonomists if possible (see acknowledgements). For the hosts that produced parasitoids the identification of similar adult lepidopterans collected at the same time were assumed to be the host for that parasitoid (this was cross-checked using the subsequent parasitoid identification). Males of certain species can be difficult to assign and in some cases we assigned a genus based on the female parasitoids that had been collected at the same time in the same location. If no females were reared then the sub-family identification or morphospecies code was used.

To supplement the herbivore-parasitoid interactions identified during the field work we collated information from the published literature. All herbivore species collected in the field were listed and the genus and species names used in an ISI Web of Knowledge^SM^ search. The search results were restricted to parasitoid species that are known to attack larval stages of the host, and are likely to be present in temperate climates in south-eastern Australia. An honours thesis [18] and hard copy taxonomic publications (e.g. [19]) were searched. We did not cite all references for each herbivore-parasitoid interaction, but collated as many unique interactions as possible.

### Malaise Trapping

We quantified the degree of insect movement across edges in the Cootamundra landscape to characterise the relative permeability of these edges. We chose three edge-types: canola adjacent to canola (the control edge), canola adjacent to cereal, and canola adjacent to NPV (Table S1 in File S1). Twelve bi-directional malaise traps (4 replicates per edge-type) were placed within the 10 km diameter circle sample area (nearest traps were 382 m apart, furthest 5.13 km apart). Each edge-type replicate was spatially independent (i.e. no field edge shared traps). Each trap resembled a small white tent (dimensions 170 cm height at front, 170 cm length, 96 cm height at back) made out of fine mesh material. One end was held up with a tent pole and had two collection bottles filled with 70% ethanol (∼250 ml) and ∼5 ml of detergent. The black mid-vein of the tent functioned as an interception trap positioned parallel to the interface. Insects flying from one direction hit the mesh and climbed upwards entering the collection bottle, while insects flying from the other direction were captured in the bottle on the opposite side. The trap catch represents insects moving from one habitat patch to another, and is not a reflection of the abundance in each habitat-patch. The traps were inspected and bottles changed weekly throughout the canola growing season. The catch from the 24 bottles for six sample weeks was used in analyses (early season: 21/05/2009, 11/06/2009, 18/06/2009, 25/06/2009; late season: 22/09/2009, 6/10/2009). Vegetation around the base of the trap was mown during the sampling period. The samples were sieved through a fine mesh strainer (0.5 mm) and stored in vials of 80% ethanol. Insects were identified to ordinal or family level and classified into functional groups (Herbivores, Predators, and Parasitoids). Some taxa were identified to species if sufficient numbers were found in the traps. On the odd occasion (11 bottles out of 144 throughout the season) when a trap was ripped or pulled down by livestock during the sampling week the catch was excluded from the data set.

### Analysis

The movement patterns measured by the 24 bottles (12 traps×2 bottles/trap) were: canola to canola (Control) 8 bottles, from canola to cereal 4 bottles, from cereal to canola 4 bottles, from canola to NPV 4 bottles, from NPV to canola 4 bottles. An ordination approach was used to explore the differences in community composition between the different edge-types. Each bottle was classified according one of five treatments that reflected edge-type and the crop next to the bottle (Table S1 in File S1). We used non-metric multidimensional scaling (MDS) in PRIMER (v6.1.13) [20] to order the samples such that their interpoint distances reflect the pattern of variation across multiple taxa. A matrix of total abundance of each taxon (for five sample dates, the first sample was removed due to very low numbers) by bottle was calculated. The abundance counts were square-root transformed (to down-weight the highly abundant species) and a Bray Curtis (also known as Sorensen) similarity matrix was created. A randomization test was used to assess the optimal number of dimensions for MDS ordination. A permutational analysis of variance using PERMANOVA+ (v1.0.3) [21] was used to test if there were significant differences in community composition between the treatments. Type III partial sums of squares were used, with unrestricted permutation of raw data, and 9999 permutations. Pair-wise comparisons were then made between the five treatments.

A generalized linear modelling (GLM) approach in GenStat [22] was used to assess if edge-type was important for explaining the variation in the density of trap catches across time. The model being assessed included the factors sampling date (6 sampling dates, Date), edge-type (5 different treatments with an unbalanced design, Edge) and the interaction between Edge and Date. The insect counts in each bottle per week were used as the response variates. We initially used a Poisson error distribution but found unacceptably high overdispersion. The final models used a negative binomial error distribution and a log link function. For each model, the aggregation parameter (k), which specifies the amount of overdispersion, was first estimated by the Newton-Raphson method [23] then used to fit the final model. Six sampling dates were used for all functional groups and most taxa, however for some taxa we could only obtain an adequate fit using the final two end of season sampling dates (in which trap density was higher). The interaction term was dropped from the final model if extra variation it explained was not significant. The differences between Edges across the sampling dates were examined by calculating the predicted values for the five Edge means, and their standard errors, from the appropriate GLM with back-adjustment from the log scale to the original scale.

High parasitoid numbers were obtained for one late-season sample date (22/09/2009) and further analysis was conducted on parasitoid taxa collected during this week. The parasitoids were grouped into Aphidiinae (Braconidae, aphid parasitoids), Microgastrinae (Braconidae), other Braconidae, *Diplazon* sp. (Ichneumonidae), other Ichneumonidae, and other hymenopteran parasitoids. A multivariate ANOVA in GenStat [22] was used to assess if any parasitoid group showed strong patterns with respect to treatment.

## Results

We reared a total of 1583 herbivores to assess if they were parasitised. The overall parasitism rate was 19% in 2009 (762 live rearings) and 18% in 2010 (821 live rearings). A greater number and diversity of parasitoid morphospecies were reared from hosts collected in the native vegetation patches than in the canola and wheat fields (Table 1, Table S2 in File S1). Only 12 of the 60 morphospecies identified were collected from more than one habitat-type. For example, the ichnuemonid ?*Campoletis* sp. B was collected in canola, wheat, pastures and NPV. Others, such as *Diadegma* sp. (collected in the canola and pasture), and *Temelucha* sp. (collected in pastures and NPV), were present in two habitats. The results were the opposite when examining the species interactions recorded in the literature (Tale S3 in file S1). The herbivore taxa were classed as either pests of grains cropping systems or non-pest. Of the 40 non-pest taxa, 31 had no recorded parasitoids, and the remaining nine had 42 unique herbivore-parasitoid interactions (mean 4.7 unique interactions per host). In comparison, of the 18 pest taxa, 3 had no recorded parasitoids, and the remaining 15 had 110 unique herbivore-parasitoid interactions (mean 7.3 unique interactions per host).

**Table 1:** Parasitoid morphospecies abundance reared from lepidopteran herbivores (larval stages only) collected from multiple habitats in mixed grain cropping landscapes.

Family	Subfamily	Morphospecies		Canola	Wheat	Pasture	Fallow	NPV^1^
Braconidae	Cheloninae	*Chelonus (Microchelonus)* sp. msp7				2		1
Braconidae	Agathidiinae	*Therophilus* spp. msp5				1		4
Braconidae	Microgastrinae	*Apanteles* sp. msp8				1	2	3
Braconidae	Microgastrinae	Micro (*Cotesia*?) msp16				1		5
Braconidae	Microgastrinae	*Microplitis* sp. mps12				2		2
Ichneumonidae	Campopleginae	?*Campoletis* sp. B		1	3	13		15
Ichneumonidae	Campopleginae	*Diadegma* sp.		4		1		
Ichneumonidae	Campopleginae	*Eucaphila vulgaris*			1			1
Ichneumonidae	Cremastinae	*Temelucha* sp.				1		3
Ichneumonidae		Ichneumonidae unknown				2		5
Eulophidae	Elasminae	*Elasmus* sp.					3	7
Tachinidae	Exoristinae	*Exorista* msp1				1		2
Number of additional morphospecies unique to each habitat				2	3	11	2	30

Rearing data from two years combined. Only morphospecies collected from multiple habitats are shown (those unique to each habitat are detailed in Table S2 in File S1).

1. NPV = native perennial vegetation

**Figure 1:**
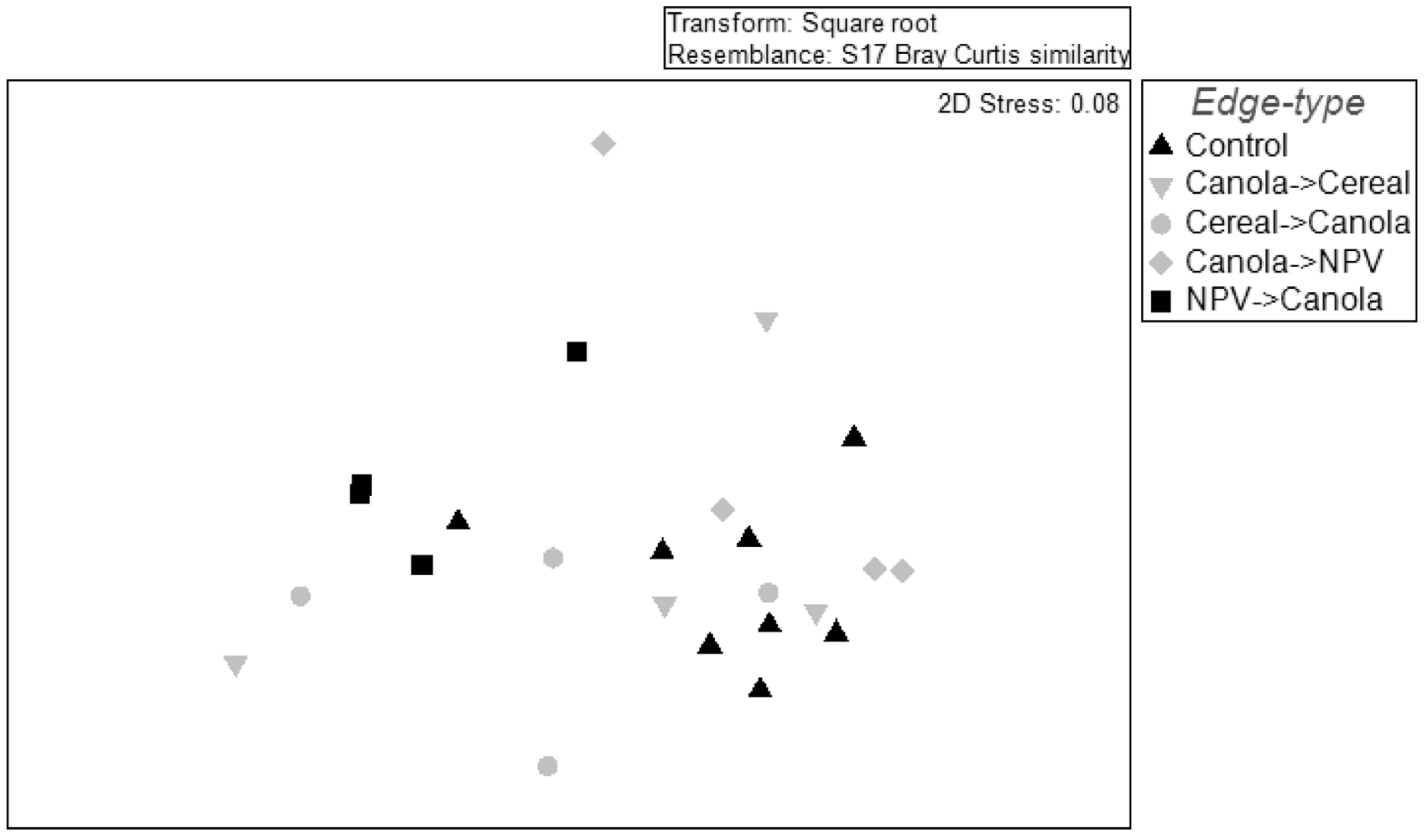
Ordination (non-metric multi-dimensional scaling) of communities found in bi-directional malaise traps positioned on different edge-types. Data set included the total abundance of 16 arthropod taxa collected across five sampling weeks (3 early season and 2 late season) found in 24 sampling bottles. Black triangles show the community moving across the canola/canola control edge, and black squares the community moving from native perennial vegetation patches (NPV) into canola fields (Can).

The insects captured in the bi-directional malaise traps showed clear differences in how they perceive the degree of contrast between the habitats. The ordination results showed that there were significant differences between the insect communities found at the different edges. The ordination diagram (Fig. 1) shows that samples moving from NPV are arranged in a different ordination space to the control samples. The PERMANOVA results suggest an overall significant difference in the community composition between the different edge-types (Pseudo *F*_4,19_ = 2.16, *P(perm)* = 0.027). The biggest differences were observed between either side of the NPV trap (canola to NPV and NPV to canola; *t* = 2.20, *P(perm)* = 0.031), and the insects moving out of a NPV into canola in comparison to the control edge (control and NPV to canola; *t* = 2.77, *P(perm)* = 0.005).

**Figure 2:**
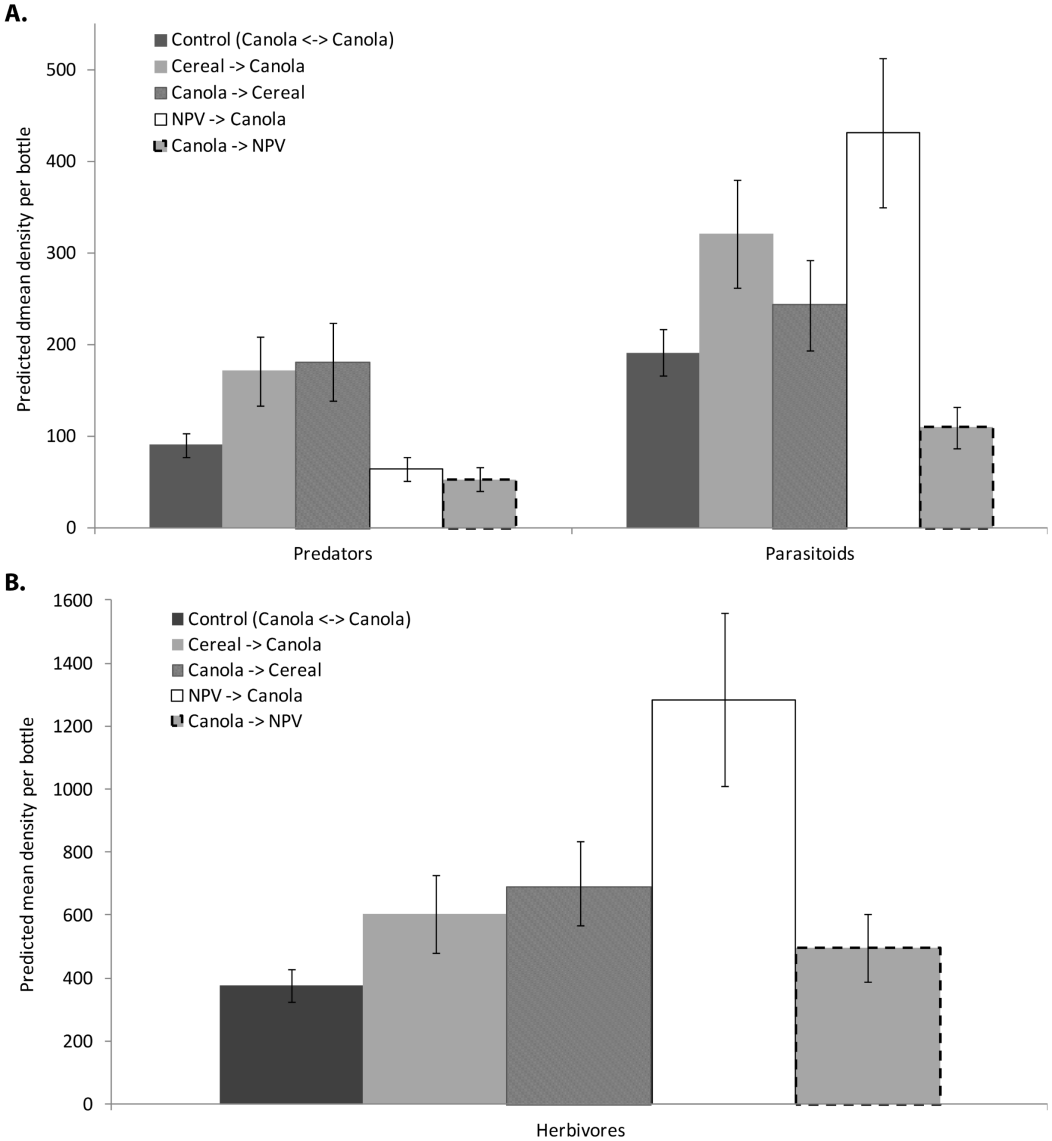
Predicted values from GLM analysis showing density of insects moving across different edge-types. Data collected from bi-directional malaise traps positioned on three edge-types in a mixed grain cropping landscape. Insects were grouped into predator, parasitoid (A) and hebivore (B) functional groups. Canola was the target crop and the controls were samples from canola adajcent to canola edge, compared to canola adjacent to cereal edge, and canola adjacent to NPV (native perennial vegetation). GLM were used to test for edge-effects (see Table 2). Data were back-transformed to the original scale and bars indicate one standard error.

Analysis of insect captures in the 133 samples from the bottles in the malaise traps showed that total insects captured increased as the season progressed (range of mean per bottle 734±85 s.e. per week in early June, to 3500±380 in late September) with greater numbers of all taxa in the two late season samples. Unsurprisingly, sample date was consistently significant (except for Thysanoptera, Table 2). Edge-type was an important factor for explaining the variability in trap catch density for all functional groups (Fig. 2). There was a strong interaction between edge-type and sample date for all functional groups except predators (which had a weak interaction *P* = 0.051). Lepidopteran moths, Syrphidae and Coccinellidae did not show a significant edge-effect, but the moths and Coccinellidae did show some interaction between edge and date. Not all taxa followed the hypothesised trend of predators and parasitoids moving out of the NPV into crops at a greater rate than across the control edge (Table 2). For predators the NPV was the least common source of individuals moving into canola fields, and cereal the most common in both early and late season traps (Fig. 2). Neuroptera and Coleopteran predators moved more commonly from cereal fields in late season samples. For both parasitoids and herbivores (Table 2) there was a change in source area from cereal fields early season, to NPV late in the season. The herbivore functional group was dominated by aphids in late season samples and showed the same relative rank of source areas, with greater densities moving from NPV (Table 2, Fig. 2).

**Table 2:** Movement across different edge-types by insect taxa in an agricultural landscape.

	Edge-type	Interaction	k^1^	RankEarly season	RankLate season
Models with data from all 6 dates					
**Total**	<0.001	<0.001	5.32	NPV>can>cer	NPV>cer>can
**Predators**	<0.001	0.051	3.50	cer>can>NPV	cer>can>NPV
**Parasitoids**	<0.001	0.009	3.40	cer>can>NPV	NPV>cer>can
**Herbivores**	0.028	<0.001	4.52	cer>can>NPV	NPV>cer>can
**Aphididae**	<0.001	0.024	1.75	NPV>can>cer	NPV>cer>can
**Coleopteran herbivores**	<0.01	0.034	2.48	cer>NPV>can	cer>NPV>can
**Hemipteran herbivores**	<0.001	–	3.96	NPV>cer>can	
**Lepidopteran moths**	0.244	0.005	6.16	can>cer>NPV	NPV>can>cer
**Neuroptera**	0.038	0.033	3.56	can>NPV>cer	cer>can>NPV
Models with data from 2 late season dates					
**Thysanoptera**	0.008	0.033	0.71	Low density	can>NPV>cer
**Syrphidae**	0.243	–	2.06	Low density	No difference
**Vespoidea**	<0.001	0.052	1.50	Low density	cer>NPV>can
**Coleopteran predators**	<0.001	–	0.75	Low density	cer>can>NPV
**Coccinellidae**	0.093	0.019	1.48	Low density	No difference

Table shows the results of GLM using Edge-type and sampling Date as explanatory variables. The counts of insects collected in each side of a bi-directional malaise trap per week were used as the response variable. For all taxa Date was significant (P<0.001 for 6 dates, P<0.05 for 2 dates), except for Thysanoptera (P = 0.278), so this term was excluded from the table. A dash indicates that the interaction term was removed from the final model. The final columns illustrate the relative rank of each source area (can = canola, cer = cereal, NPV = native perennial vegetation) from highest to lowest movement of insects.

1 Estimated aggregation parameter

**Figure 3:**
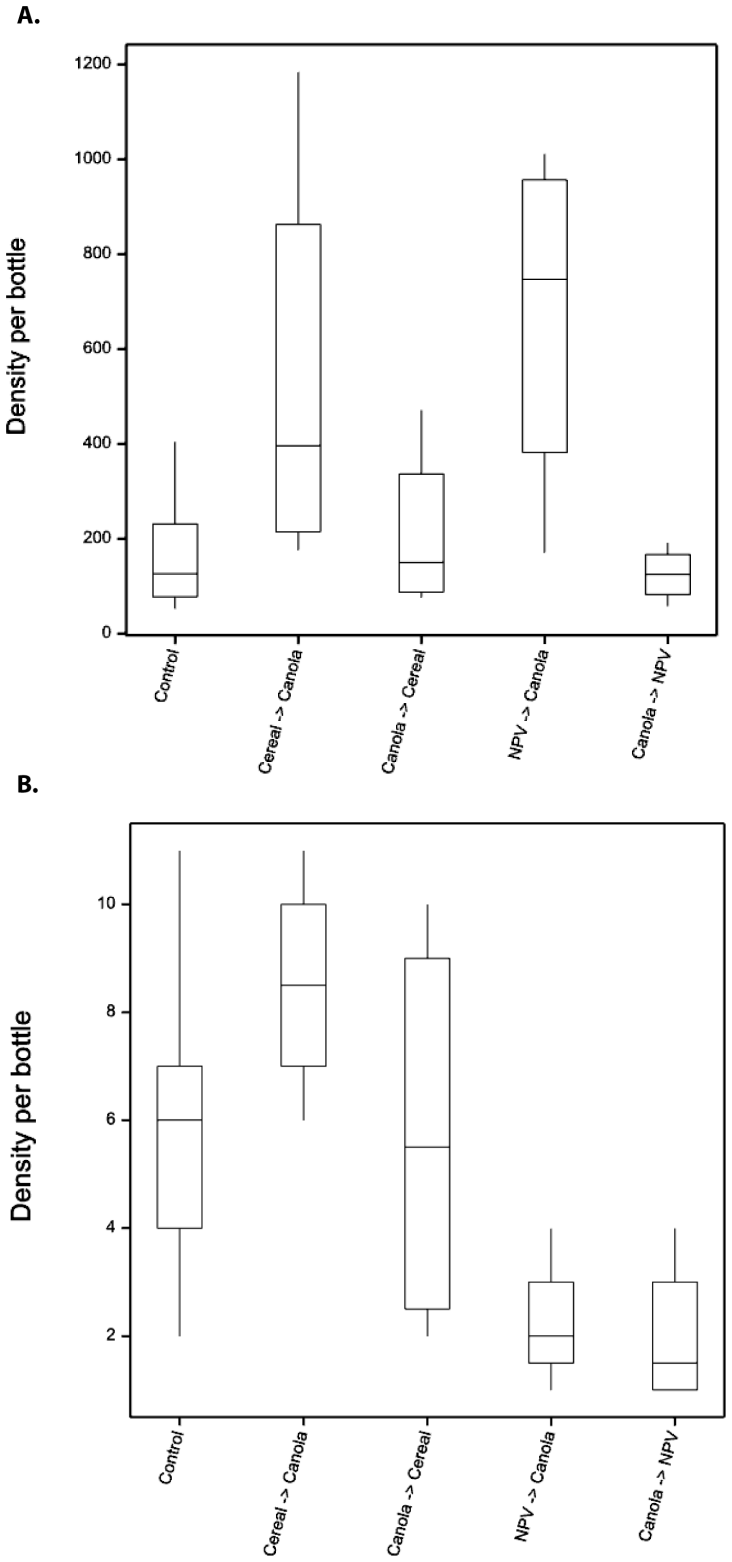
Density of parasitic wasps moving across different edge-types. Aphidiinae parasitoids (A) and Micrograstrinae parasitoids (Braconidae) (B) were captured in bi-directional malaise traps positioned on different edge-types, open for a single week late in the season. Canola was the target crop and the controls were samples from canola adjacent to canola edge, compared to canola adjacent to cereal edge, and canola adjacent to NPV (native perennial vegetation). The box spans the interquartile range of the values, the line indicates the median, whiskers indicate the minimum and maximum values.

The parasitoids moved more commonly out of cereal and out of NPV into canola than in the opposite direction, and higher than the control (Fig. 2). However, there were different patterns between parasitoid taxa. The Microgastrinae parasitoids clearly move from cereal fields into canola more often than across the control edge (Fig. 3, MANOVA: *F*_4,19_ = 5.97, *P* = 0.003), however the Aphidiinae parasitoids showed greater movement from NPV into canola (Fig. 3, MANOVA: *F*_4,19_ = 4.07, *P* = 0.015). All other parasitoid taxa showed no significant difference between edge-types.

## Discussion

### Do Native Vegetation Patches Provide Alternative Non-pest Hosts for Important Parasitoids?

There are many ways in which NPV may potentially improve productivity and environmental sustainability of farms. If NPV provides alternate hosts for parasitoid species we may see greater population abundance of parasitoid species across a landscape, and/or greater number of pests parasitised in nearby crop fields. In this study we found a good diversity of parasitoid species using hosts in NPV patches, however we recorded few instances of cross-over with species using hosts in both NPV patches and crop fields (Table 1). This is not to say such cross-overs could not occur. The literature review highlighted parasitoid species such as *Brachymeria phya* that have been recorded attacking *Plutella xylostella* (diamond-back moth) on canola and *Merophyas divulsana* (lucerne leaf roller on pastures). Other common non-pest herbivores (e.g. *Uraba lugens*, gum-leaf skeletonizer) have a large number of parasitoid species attacking them, and could potentially lead to cross-overs in certain years (Table S3 in File S1). We can use the malaise trap data to explore if the lack of cross-overs is due to a lack of movement between these patches (i.e. parasitoids in NPV only attack hosts in NPV, and those in crop fields only attack hosts in crop fields, with little movement between patches).

### Do Natural Enemies Move From Native Vegetation Patches into Adjacent Crop Fields?

The malaise trap results showed that there were significant differences in the numbers of insects moving across the different edge-types and that changed across time, with numbers increasing as the season progressed (Table 2). We expected to see greater movement of natural enemies from NPV into canola than in comparison to the control edge, if they were using alternate hosts and prey in the NPV patch. However, this pattern was not consistently found across all natural enemy functional groups or taxa studied (Table 2, Fig. 2). Lower densities of predator taxa were found moving from NPV into canola than across the control edge. For parasitoid taxa greater densities were found moving from NPV into canola, however there were similar (but slightly lower) numbers moving from cereal fields into canola. When we looked in more detail at two parasitoid taxa (Fig. 3), it was clear that these two groups differ in their use of NPV patches late in the season. Herbivores also moved frequently out of NPV (Fig. 2) and this pattern was driven by the movement of aphids late in the season. This result may be due to a number of factors, including the proportion of habitat specialists versus generalists [24] in each functional group, and the dispersal abilities of the taxon involved.

Pest control services rely on the ability of natural enemies to move frequently into crop fields and once there attack and kill pest species. In this study we examined both movement patterns and species interactions to investigate the role native vegetation in pest control services. The malaise trap data suggest that Microgastrinae parasitoids readily move from cereal fields into canola (Fig. 2), yet relatively few taxa were reared from these habitats. There are two possible explanations for the lack of concordance between the species interactions and movement data. Firstly, parasitoids are using hosts in multiple habitat patches across the landscape but our sampling strategy failed to record these interactions. For example, we did not sample herbivores in the tree canopy and this could be a large source of potential alternate hosts. Important interactions can be easily missed if host density is low, and many interactions are likely to be sporadic across space and time. Rearing specimens for more than two field seasons may be required to record some of these interactions. Secondly, the parasitoids collected in the malaise traps are moving through these habitat patches but are not using alternate host resources. They may be using other patches for floral resources or places of refuge, but only using hosts in the crop fields [25], [26].

Rearing of herbivore hosts provided some interesting species interactions that had not previously been recorded in the literature. For example, we reared a specimen that is likely to be *Taxoneuron nigriceps* (Braconidae, Cardiochilinae) from *Helicoverpa punctigera*. This species has been recorded parasitising *Heliothis virescens* and *H. zea* in the United States, and has been introduced and recorded attacking *H. assulta* and *H. armigera* in Asia (see [27] and references within), yet has not been documented as attacking *Helicoverpa* species in Australian grain crops. Conversely, there are many species of parasitoids that were documented in the literature that were not recorded throughout this study (Table S3 in File S1). The lack of overlap between this study and the literature suggests that parasitoids of pest species in grains cropping landscapes are generally not well characterised. Greater taxonomic effort is needed to clarify the spatial and temporal distribution of important species interactions.

The use of directional traps to understand movement between habitat-patches has been relatively under-utilised (the few exceptions are [3], [28]). Whilst malaise traps are useful for examining movement patterns, translating these into abundance estimates in the crop is difficult. Some species have shown a positive response to edges such that their abundance or activity is increased at the interface [24], [29] and may not reflect the levels observed further into the crop field. The traps give us a good indication of which taxa are moving between different components of the landscape and the functional connectivity of these patches [2]. However, our insights are limited only to insects that are strong flyers and exclude some taxa that may be able to avoid the traps (e.g. some bee species). The wind strength and direction may contribute to variability in trap catch, however given the traps are close to the ground and capturing insects for 7 days, the effect would be difficult to quantify.

Quantifying how species perceive habitat patches and matrix in agricultural landscapes is a challenge [2], [30]. Movement patterns can help us to identify patches that act as a source for species at particular times during the season. The taxa studied here displayed different response to edge-type and some were less likely to move across edges. How this relates to the role of NPV in the provision of pest control services is difficult to judge. At the landscape-scale there is evidence that the configuration and quantity of non-crop vegetation can impact pest control services [13], [31]. In this study we found that alternate hosts in NPV represent a potential resource for parasitoids, however we also saw large numbers of parasitoids moving from cereal fields into canola fields. In contrast, some predatory species such as Neuropterans, appear to be true habitat generalists and are able to move and utilise resources across a range of habitats. Examining how populations of such species respond if all NPV was removed from a cropping landscape would be the next step in assessing the true value of these patches.

## Supporting Information

File S1 Tables providing additional details on the treatments used during the sampling (Table S1), the complete list of all species interactions recorded (Table S2) and a complete list of the species interactions recorded in the literature (Table S3).
(DOCX)
