# Supplementary material for: Edges in Agricultural Landscapes: Species Interactions and Movement of Natural Enemies
Source: PLoS One. 2013 Mar 26;8(3):e59659. doi: 10.1371/journal.pone.0059659 (PMC3608671; doi:10.1371/journal.pone.0059659)
Supplement: File S1 — Tables providing additional details on the treatments used during the sampling (Table S1), the complete list of all species interactions recorded (Table S2) and a complete list of the species interactions recorded in the literature (Table S3). (DOCX) [file pone.0059659.s001.docx]

**SUPPORTING INFORMATION**

**Table S1.** Summary of edge-types selected for malaise trapping. NPV = native perennial vegetation patch

| Edge type | Crop next to bottle | Treatment code | Number of bottles per week |
| --- | --- | --- | --- |
| Canola/Canola | Canola | Control | 8 |
| Canola/Cereal | Canola | From canola to cereal | 4 |
| Canola/NPV | Canola | From canola to NPV | 4 |
| Canola/Cereal | Cereal (wheat or barley) | From cereal to canola | 4 |
| Canola/NPV | NPV | From NPV to canola | 4 |

**Table S2.** Parasitoid morphospecies reared from lepidopteran herbivores (larval stages only) collected from multiple habitats in mixed grain cropping landscapes. Rearing data from two year combined (2009 and 2010).

| Family | Subfamily | Morphospecies identification | Canola | Wheat | Pasture | Fallow arable field | NV |
| --- | --- | --- | --- | --- | --- | --- | --- |
| Braconidae | Cheloninae | *Chelonus (Microchelonus)* sp. msp7 |  |  | 2 |  | 1 |
| Braconidae | Agathidiinae | *Therophilus* spp. msp5 |  |  | 1 |  | 4 |
| Braconidae | Agathidiinae | *Therophilus* spp. msp6 |  |  |  |  | 2 |
| Braconidae | Rogadinae | *Aleiodes*? msp1 |  |  |  |  | 7 |
| Braconidae | Rogadinae | Rogadinae msp2 |  |  |  |  | 1 |
| Braconidae | Rogadinae | Rogadinae msp4 |  |  |  |  | 1 |
| Braconidae | Cardiochilinae | *Toxoneuron nigriceps*? msp9 |  |  | 1 |  |  |
| Braconidae | Microgastrinae | *Apanteles* sp. msp8 |  |  | 1 | 2 | 3 |
| Braconidae | Microgastrinae | *Glyptapanteles* sp1 msp10 |  |  |  |  | 2 |
| Braconidae | Microgastrinae | *Glyptapanteles* sp2 msp11 |  |  | 1 |  |  |
| Braconidae | Microgastrinae | *Glyptapanteles* sp3? msp13 |  |  |  |  | 1 |
| Braconidae | Microgastrinae | Micro (*Cotesia*?) msp14 |  |  | 2 |  |  |
| Braconidae | Microgastrinae | Micro (*Cotesia*?) msp15 |  | 1 |  |  |  |
| Braconidae | Microgastrinae | Micro (*Cotesia*?) msp16 |  |  | 1 |  | 5 |
| Braconidae | Microgastrinae | Micro unknown |  |  | 1 |  |  |
| Braconidae | Microgastrinae | *Microplitis* sp. mps12 |  |  | 2 |  | 2 |
| Braconidae | Rogadinae | *Orgilus* sp.msp3 |  |  |  |  | 1 |
| Braconidae |  | Braconidae unknown |  |  |  | 8 |  |
| Ichneumonidae | Campopleginae | ?*Campoletis* sp. A |  |  | 2 |  |  |
| Ichneumonidae | Campopleginae | ?*Campoletis* sp. A |  |  | 1 |  |  |
| Ichneumonidae | Campopleginae | ?*Campoletis* sp. B | 1 | 3 | 13 |  | 15 |
| Ichneumonidae | Banchinae | Banchinae unknown |  |  |  |  | 2 |
| Ichneumonidae | Campopleginae | Campopleginae sp. A |  |  | 2 |  |  |
| Ichneumonidae | Campopleginae | Campopleginae sp. B |  | 1 |  |  | 1 |
| Ichneumonidae | Campopleginae | Campopleginae sp. C |  |  |  |  | 1 |
| Ichneumonidae | Campopleginae | Campopleginae sp. D |  |  | 1 |  |  |
| Ichneumonidae | Campopleginae | Campopleginae sp. E | 1 |  |  |  |  |
| Ichneumonidae | Campopleginae | Campopleginae sp. F |  | 1 |  |  |  |
| Ichneumonidae | Tryphoninae | *Phytodietus* (Weisia) sp. |  |  |  |  | 1 |
| Ichneumonidae | Campopleginae | *Casinaria* sp. |  |  |  |  | 13 |
| Ichneumonidae | Campopleginae | *Delopia* sp. |  |  |  |  | 1 |
| Ichneumonidae | Campopleginae | *Diadegma* sp. | 4 |  | 1 |  |  |
| Ichneumonidae | Campopleginae | *Eriborus* sp. A |  |  |  |  | 3 |
| Ichneumonidae | Campopleginae | *Eriborus* sp. B |  |  | 1 |  |  |
| Ichneumonidae | Campopleginae | *Eucaphila vulgaris* |  | 1 |  |  | 1 |
| Ichneumonidae | Banchinae | *Glypta ?rufiscutellaris* |  |  |  |  | 2 |
| Ichneumonidae | Banchinae | *Lissonota* sp. |  |  |  |  | 1 |
| Ichneumonidae | Mesochorinae | *Mesochorus* sp. |  |  |  |  | 8 |
| Ichneumonidae | Cremastinae | *Pristomerus* sp. |  |  |  |  | 1 |
| Ichneumonidae | Cremastinae | *Temelucha* sp. |  |  | 1 |  | 3 |
| Ichneumonidae |  | Ichneumonidae unknown |  |  | 2 |  | 5 |
| Chalcididae | Brachymeriinae | *Brachymeria* sp. |  |  |  | 3 |  |
|  |  | Chalcididoidea unknown |  |  |  |  | 1 |
| Encyrtidae | Encyrtinae | *Copidosoma*-like msp1 |  |  |  |  | 2 |
| Eulophidae |  | Eulophidae unknown |  |  |  |  | 2 |
| Eulophidae | Entedoninae | *Asecodes* msp1 |  |  |  |  | 3 |
| Eulophidae | [Eulophinae](http://www.ento.csiro.au/science/Liriomyza_ver3/key/Liriomyza_Parasitoids_Key/Media/Html/eulophinae.html) | *Cirrospilus* msp1 |  |  |  |  | 1 |
| Eulophidae | [Eulophinae](http://www.ento.csiro.au/science/Liriomyza_ver3/key/Liriomyza_Parasitoids_Key/Media/Html/eulophinae.html) | *Cirrospilus* msp2 |  |  |  |  | 5 |
| Eulophidae | [Eulophinae](http://www.ento.csiro.au/science/Liriomyza_ver3/key/Liriomyza_Parasitoids_Key/Media/Html/eulophinae.html) | *Cirrospilus* msp3 |  |  |  |  | 2 |
| Eulophidae | [Eulophinae](http://www.nhm.ac.uk/research-curation/research/projects/chalcidoids/database/detail.dsml?FamilyCode=HE&ValFamTrib=Eulophinae) | *Diaulomorpha* msp1 |  |  |  |  | 5 |
| Eulophidae | Elasminae | *Elasmus* sp. |  |  |  | 3 | 7 |
| Perliampidae |  | *Perilampos* msp1 |  |  |  |  | 2 |
| Torymidae |  | Torymidae unknown |  |  |  |  | 1 |
| Tachinidae | Exoristinae | *Calozenillia* msp1 |  |  |  |  | 1 |
| Tachinidae | Exoristinae | *Carcelia* msp1 |  |  |  |  | 2 |
| Tachinidae |  | *Chaetophthalmus* msp1 |  |  | 1 |  |  |
| Tachinidae | [Exoristinae](http://en.wikipedia.org/wiki/Exoristinae) | *Exorista* msp1 |  |  | 1 |  | 2 |
| Tachinidae | [Exoristinae](http://en.wikipedia.org/wiki/Exoristinae) | *Goniophthalmus* msp1 |  |  | 3 |  |  |
| Tachinidae | [Exoristinae](http://en.wikipedia.org/wiki/Exoristinae) | *Sisyropa* msp1 | 1 |  |  |  |  |
| Tachinidae | [Exoristinae](http://en.wikipedia.org/wiki/Exoristinae) | Winthemia msp1 |  |  |  |  | 1 |
| Tachinidae | Tachininae | *Siphonini* unknown msp1 |  | 1 |  |  |  |
| Total abundance |  |  | 7 | 8 | 41 | 16 | 125 |
| Number of morphospecies | |  | 4 | 6 | 21 | 4 | 42 |

**Table S3.** Herbivore-parasitoid interactions recorded from the literature. Host species list was generated via rearing herbivores from a mixed grain cropping landscapes. Host species was used as the primary search term. A species was considered a pest species if it was recorded attacking grains crops or pasture species in south-east Australia. Only parasitoids attacking larval stages of the host were included.

| Host | Pest species | Parasitoid | Family | Reference^2^ |
| --- | --- | --- | --- | --- |
| *Achyra affinitalis* | No | *Temelucha cycnea* | Ichneumonidae | Gauld 1984[1] |
| *Acropolitis excelsa* | No |  |  |  |
| *Acyphas* sp. | No | *Carcelia vicinalis* | Tachinidae | Cantrell 1986[2] |
| *Acyphas* sp. | No | *Delopia* sp. | Ichneumonidae | Gauld 1984[1] |
| *Acyphas* sp. | No | *Enicospilus sausi* | Ichneumonidae | Gauld 1984[1] |
| *Acyphas* sp. | No | *Hyposoter* sp. | Ichneumonidae | Gauld 1984[1] |
| *Agrophara* sp. | No |  |  |  |
| *Aproaerema isoscelixantha* | No |  |  |  |
| *Ardozyga stratifera* | No | *Diaulomorpha sp1.* | Eulophidae | Gamez-Virues *et al.*  2009[3] |
| *Ardozyga stratifera* | No | *Glyptapanteles* sp. | Braconidae | Gamez-Virues *et al.*  2009[3] |
| *Ardozyga stratifera* | No | *Neostomatoceras* sp. | Chalcididae | Gamez-Virues *et al.*  2009[3] |
| *Ardozyga stratifera* | No | *Sierola* sp. | Bethylidae | Gamez-Virues *et al.*  2009[3] |
| *Circopetes obtusata* | No |  |  |  |
| *Crocidosema plebejana* | No |  |  |  |
| *Crypsiphona occultaria* | No |  |  |  |
| *Dasygaster* sp. | No |  |  |  |
| *Dialectica scalariella* | No | *Stenomesius japonicus* | Eulophidae | James & Stevens 1992[4] |
| *Dissomorphia australiaria* | No |  |  |  |
| *Euchaetis* | No |  |  |  |
| *Eupselia beltera* | No |  |  |  |
| *Eupselia satrapella* | No |  |  |  |
| *Gastrinodes argoplaca* | No |  |  |  |
| *Holocola* sp. | No |  |  |  |
| *Hyalarcta huebneri* | No |  |  |  |
| *Hyalarcta nugrescens* | No |  |  |  |
| *Hypertropha* sp. | No |  |  |  |
| *Isturgia penthearia* | No |  |  |  |
| *Macrobathra* sp. | No | *Temelucha minuta* | Ichneumonidae | Gauld 1984[1] |
| *Microdes squamulata* | No | *Phytodietus* sp. | Ichneumonidae | Gauld 1984[1] |
| *Morosaphycita oculiferella* | No |  |  |  |
| *Neumichtis nigerrima* | No |  |  |  |
| *Notodryas* sp. | No |  |  |  |
| *Oenochroa* sp. | No |  |  |  |
| *Pantydia sparsa* | No | *Tricholabus sp1.* | Ichneumonidae | Gauld 1984[1] |
| *Pararguda nasuta* | No |  |  |  |
| *Pataeta* sp. | No |  |  |  |
| *Perthida* sp. | No |  |  |  |
| *Phrissogonus laticostata* | No |  |  |  |
| *Proteuxoa oxygona* | No |  |  |  |
| *Proteuxoa tibiata* | No |  |  |  |
| *Strepsicrates macropetana* | No | *Trigonospila brevifacies* | Tachinidae | internet^1^ |
| *Strepsicrates macropetana* | No | *Xanthopimpla rhopaloceros* | Ichneumonidae | internet^1^ |
| *Syringoseca mimica* | No |  |  |  |
| *Tebenna micalis* | No |  |  |  |
| *Tymbophora peltastis* | No |  |  |  |
| *Uraba lugens* | No | *?Tetrastichus Haliday* sp. | Eulophidae | Berndt & Allen 2010[5] |
| *Uraba lugens* | No | *Anacis* sp. | Ichneumonidae | Mansfield *et al.*  (2005)[6] |
| *Uraba lugens* | No | *Anastatus* sp. | Eupelmidae | Berndt & Allen 2010[5] |
| *Uraba lugens* | No | *Antrocephalus* sp. | Chalcididae | Berndt & Allen 2010[5] |
| *Uraba lugens* | No | *Brachymeria froggatti* | Chalcididae | Berndt & Allen 2010[5] |
| *Uraba lugens* | No | *Brachymeria rubripes* | Chalcididae | Berndt & Allen 2010[5] |
| *Uraba lugens* | No | *Brachymeria* sp. *1* | Chalcididae | Berndt & Allen 2010[5] |
| *Uraba lugens* | No | *Brachymeria* sp. *2* | Chalcididae | Berndt & Allen 2010[5] |
| *Uraba lugens* | No | *Campoplex* sp. | Ichneumonidae | Gauld 1984 |
| *Uraba lugens* | No | *Campyloneura* sp. | Braconidae | Berndt & Allen 2010[5] |
| *Uraba lugens* | No | *Centrodora* sp. | Aphelenidae | Berndt & Allen 2010[5] |
| *Uraba lugens* | No | *Cotesia urabae* | Braconidae | Berndt & Allen 2010[5] |
| *Uraba lugens* | No | *Dolichogenidea eucalypti* | Braconidae | Berndt & Allen 2010[5] |
| *Uraba lugens* | No | *Elasmus australiensis* | Elasmidae | Berndt & Allen 2010[5] |
| *Uraba lugens* | No | *Elasmus* sp. | Elasmidae | Berndt & Allen 2010[5] |
| *Uraba lugens* | No | *Euplectrus* sp. | Eulophidae | Berndt & Allen 2010[5] |
| *Uraba lugens* | No | *Eurytoma* sp. | Eulophidae | Berndt & Allen 2010[5] |
| *Uraba lugens* | No | *Exorista flaviceps* | Tachinidae | Berndt & Allen 2010[5] |
| *Uraba lugens* | No | *Irabatha* sp. | Braconidae | Berndt & Allen 2010[5] |
| *Uraba lugens* | No | *Meteorus pulchricornis* | Braconidae | Mansfield *et al.*  (2005)[6] |
| *Uraba lugens* | No | *Pediobus bruchicida* | Eulophidae | Berndt & Allen 2010[5] |
| *Uraba lugens* | No | *Pediobus* sp. | Eulophidae | Berndt & Allen 2010[5] |
| *Uraba lugens* | No | *Pristomerus* sp. *4* | Ichneumonidae | Gauld 1984[1] |
| *Uraba lugens* | No | *Stiromesostenus spp.* | Ichneumonidae | Gauld 1984[1] |
| *Uraba lugens* | No | *Winthemia lateralis* | Tachinidae | Berndt & Allen 2010[5] |
| *Uraba lugens* | No | *Xanthopimpla rhopaloceros* | Ichneumonidae | Gauld 1984[1], Mansfield *et al.*  (2005)[6] |
| *Agrotis infusa* | Yes | *Chaetophthalmus bicolor* | Tachinidae | Cantrell 1986[2] |
| *Agrotis infusa* | Yes | *Netelia producta* | Ichneumonidae | Gauld 1984[1] |
| *Chrysodeixis argentifera* | Yes | *Carcelia illota* | Tachinidae | Cantrell 1986[2] |
| *Chrysodeixis argentifera* | Yes | *Ceromya* sp. | Tachinidae | Cantrell 1986[2] |
| *Chrysodeixis argentifera* | Yes | *Exorista psychidivora* | Tachinidae | Cantrell 1986[2] |
| *Chrysodeixis argentifera* | Yes | *Microplitis demolitor* | Braconidae | Austin & Dangerfield 1993[7] |
| *Chrysodeixis argentifera* | Yes | *Microplitis murrayi* | Braconidae | Austin & Dangerfield 1993[7] |
| *Chrysodeixis argentifera* | Yes | *Palexorista* sp. | Tachinidae | Cantrell 1986[2] |
| *Ciampa arietaria* | Yes |  |  |  |
| *Epiphyas postvittana* | Yes | *Actia* sp. | Tachinidae | Cantrell 1986[2] |
| *Epiphyas postvittana* | Yes | *Apanteles* sp. | Braconidae | Common 1990 p59.[8] |
| *Epiphyas postvittana* | Yes | *Australoglypta latrobei* | Ichneumonidae | Gauld 1984[1], Paull & Austin 2006[9] |
| *Epiphyas postvittana* | Yes | *Bassus* sp. | Braconidae | Paull & Austin 2006[9] |
| *Epiphyas postvittana* | Yes | *Dolichogenidea tasmanica* | Braconidae | Paull & Austin 2006[9] |
| *Epiphyas postvittana* | Yes | *Euceros* sp. | Ichneumonidae | Paull & Austin 2006[9] |
| *Epiphyas postvittana* | Yes | *Eupsenella* sp. | Bethylidae | Paull & Austin 2006[9] |
| *Epiphyas postvittana* | Yes | *Exochus* sp. | Ichneumonidae | Common 1990 p59.[8] |
| *Epiphyas postvittana* | Yes | *Goniozus mandibulatus* | Bethylidae | Paull & Austin 2006[9] |
| *Epiphyas postvittana* | Yes | *Isdromas* sp. *14* | Ichneumonidae | Gauld 1984[1] |
| *Epiphyas postvittana* | Yes | *Oedemopsis hobartensis* | Ichneumonidae | Gauld 1984[1] |
| *Epiphyas postvittana* | Yes | *Phytodictus* sp. | Ichneumonidae | Common 1990 p59.[8] |
| *Epiphyas postvittana* | Yes | *Phytodietus (Phytodietus) celsissimus* | Ichneumonidae | Gauld 1984[1] |
| *Epiphyas postvittana* | Yes | *Phytodietus celsissimus* | Ichneumonidae | Paull & Austin 2006[9], Gauld 1984[1] |
| *Epiphyas postvittana* | Yes | *Temelucha minuta* | Ichneumonidae | Paull & Austin 2006[9] |
| *Epiphyas postvittana* | Yes | *Therophilus rugosus* | Braconidae | Stevens *et al.*  2011 |
| *Epiphyas postvittana* | Yes | *Therophilus unimaculatus* | Braconidae | Stevens *et al.*  2011 |
| *Epiphyas postvittana* | Yes | *Trigonospila brevifacies* | Tachinidae | Cantrell 1986[2] |
| *Epiphyas postvittana* | Yes | *Xanthopimpla rhopaloceros* | Ichneumonidae | Gauld 1984[1] |
| *Epiphyas* sp. | Yes | *Eriborus* sp. | Ichneumonidae | Gauld 1984[1] |
| *Helicoverpa armigera* | Yes | *Carcelia cosmophilae* | Tachinidae | Cantrell 1986[2] |
| *Helicoverpa armigera* | Yes | *Chaetophthalmus dorsalis* | Tachinidae | Walker 2011[10] |
| *Helicoverpa armigera* | Yes | *Heteropelma scaposum* | Ichneumonidae | Johns & Whitehouse 2004[11] |
| *Helicoverpa armigera* | Yes | *Microplitis demolitor* | Braconidae | Austin & Dangerfield 1993 |
| *Helicoverpa armigera* | Yes | *Netelia producta* | Ichneumonidae | Johns & Whitehouse 2004[11] |
| *Helicoverpa armigera* | Yes | *Netelia spp.* | Ichneumonidae | Gauld 1984[1] |
| *Helicoverpa armigera* | Yes | *Winthemia lateralis* | Tachinidae | Cantrell 1986[2] |
| *Helicoverpa punctigera* | Yes | *Chaetophthalmus dorsalis* | Tachinidae | Walker 2011[10] |
| *Helicoverpa punctigera* | Yes | *Goniophthalmus australis* | Tachinidae | Cantrell 1986[2] |
| *Helicoverpa punctigera* | Yes | *Microplitis demolitor* | Braconidae | Austin & Dangerfield 1993[7] |
| *Helicoverpa punctigera* | Yes | *Palexorista* sp. | Tachinidae | Cantrell 1986[2] |
| *Helicoverpa spp.* | Yes | *Heteropelma scaposum* | Ichneumonidae | Johns & Whitehouse 2004[11] |
| *Helicoverpa spp.* | Yes | *Netelia producta* | Ichneumonidae | Johns & Whitehouse 2004[11] |
| *Heliothis* sp. | Yes | *Netelia producta* | Ichneumonidae | Gauld 1984[1] |
| *Leucania stenographa* | Yes |  |  |  |
| *Merophyas divulsana* | Yes | *Alophmorpha* sp. | Braconidae | Bishop & McKenzie 1991[12] |
| *Merophyas divulsana* | Yes | *Apanteles* sp. | Braconidae | Common 1990 p59.[8] |
| *Merophyas divulsana* | Yes | *Apanteles tasmanica* | Braconidae | Bishop & McKenzie 1991[12] |
| *Merophyas divulsana* | Yes | *Brachymeria phya* | Chalcididae | Bishop & McKenzie 1991[12] |
| *Merophyas divulsana* | Yes | *Bracon* sp. | Braconidae | Bishop & McKenzie 1991[12] |
| *Merophyas divulsana* | Yes | *Chelonus* sp. | Braconidae | Bishop & McKenzie 1991[12] |
| *Merophyas divulsana* | Yes | *Cotesia* sp. | Braconidae | Bishop & McKenzie 1991[12] |
| *Merophyas divulsana* | Yes | *Elasmus pictus* | Elasmidae | Bishop & McKenzie 1991[12] |
| *Merophyas divulsana* | Yes | *Eupsenella* sp. | Bethylidae | Bishop & McKenzie 1991[12] |
| *Merophyas divulsana* | Yes | *Habrobracon* sp. | Braconidae | Bishop & McKenzie 1991[12] |
| *Merophyas divulsana* | Yes | *Phytodietus* sp. | Ichneumonidae | Bishop & McKenzie 1991[12] |
| *Merophyas divulsana* | Yes | *Poecilocryptus* sp. | Ichneumonidae | Bishop & McKenzie 1991[12] |
| *Merophyas divulsana* | Yes | *Pristomerus* sp. | Ichneumonidae | Bishop & McKenzie 1991[12] |
| *Merophyas divulsana* | Yes | *Stomatomyia tricholygoides* | Tachinidae | Cantrell 1986[2] |
| *Merophyas divulsana* | Yes | *Temelucha* sp. | Ichneumonidae | Gauld 1984[1] |
| *Merophyas divulsana* | Yes | *Temelucha sp4.* | Ichneumonidae | Gauld 1980 |
| *Merophyas divulsana* | Yes | *Therophilus unimaculatus* | Braconidae | Stevens *et al.*  2011 |
| *Merophyas divulsana* | Yes | *Trichomma clavipes* | Ichneumonidae | Bishop & McKenzie 1991[12] |
| *Merophyas divulsana* | Yes | *Voriella uniseta* | Tachinidae | Bishop & McKenzie 1991[12] |
| *Merophyas divulsana* | Yes | *Xanthopimpla rhopaloceros* | Ichneumonidae | Gauld 1984[1] |
| *Mocis alterna* | Yes |  |  |  |
| *Mythimna convecta* | Yes | *Apanteles ruficrus* | Braconidae | Greenup 1970 (in Hardwick 2006[13]), Common 1990 p59.[8] |
| *Mythimna convecta* | Yes | *Ceromya horma* | Tachinidae | Hardwick 2006, Greenup 1970 (in Hardwick 2006[13]) |
| *Mythimna convecta* | Yes | *Chaetophthalmus dorsalis* | Tachinidae | Walker 2011[10] |
| *Mythimna convecta* | Yes | *Chaetophthalmus* sp. | Tachinidae | Hardwick 2006[13] |
| *Mythimna convecta* | Yes | *Cuphocera* sp. *nr pilosa* | Tachinidae | Hardwick 2006[13] |
| *Mythimna convecta* | Yes | *Hyposoter* sp. | Ichneumonidae | Gauld 1984[1] |
| *Mythimna convecta* | Yes | *Lissopimpla excellsa* | Ichneumonidae | Greenup 1970 (in Hardwick 2006[13]) |
| *Mythimna convecta* | Yes | *Microplitis murrayi* | Braconidae | Austin & Dangerfield 1993[7] |
| *Mythimna convecta* | Yes | *Netelia producta* | Ichneumonidae | Gauld 1984[1] |
| *Mythimna convecta* | Yes | *Netelia* sp. | Ichneumonidae | Hardwick 2006[13] |
| *Mythimna convecta* | Yes | *Palexorista spp.* | Tachinidae | Greenup 1970 (in Hardwick 2006[13]) |
| *Mythimna convecta* | Yes | *Rogas* sp. | Braconidae | Greenup 1970 (in Hardwick 2006[13]) |
| *Mythimna convecta* | Yes | *Tritaxys scutellate* | Tachinidae | Hardwick 2006[13] |
| *Mythimna* sp. | Yes | *Enicospilus skeltonii* | Ichneumonidae | Gauld 1984[1] |
| *Pieris rapae* | Yes | *Apanteles glomeratus* | Braconidae | Jones 1987[14], Hamilton 1979[15] |
| *Pieris rapae* | Yes | *Compsilura concinnata* | Tachinidae | Cantrell 1986[2] |
| *Pieris rapae* | Yes | *Cotesia rubecula* | Braconidae | Keller 1990[16] |
| *Pieris rapae* | Yes | *Exorista flaviceps* | Tachinidae | Rahman 1970 |
| *Pieris rapae* | Yes | *Goryphus turneri* | Ichneumonidae | Gauld 1984[1] |
| *Pieris rapae* | Yes | *Netelia producta* | Ichneumonidae | Gauld 1984[1] |
| *Pieris rapae* | Yes | *Paradrino laevicula* | Tachinidae | Cantrell 1986 |
| *Pieris rapae* | Yes | *Paraphylax* sp. *11* | Ichneumonidae | Gauld 1984[1] |
| *Pieris rapae* | Yes | *Winthemia lateralis* | Tachinidae | Cantrell 1986[2] |
| *Plutella xylostella* | Yes | *Antrocephalus* sp. | Chalcididae | Kent 1996[17] |
| *Plutella xylostella* | Yes | *Apanteles ippeus* | Braconidae | Kent 1996[17], Hamilton 1979[15] |
| *Plutella xylostella* | Yes | *Brachymeria phya* | Chalcididae | Kent 1996, Common 1990 p58, Hamilton 1979[15] |
| *Plutella xylostella* | Yes | *Cotesia plutellae* | Braconidae | Kent 1996[17] |
| *Plutella xylostella* | Yes | *Diadegma eucerophaga* | Ichneumonidae | Gauld 1984[1] |
| *Plutella xylostella* | Yes | *Diadegma fenestralis* | Ichneumonidae | Kent 1996[17] |
| *Plutella xylostella* | Yes | *Diadegma rapi* | Ichneumonidae | Kent 1996[17], Gauld 1984[1] |
| *Plutella xylostella* | Yes | *Diadegma semiclausum* | Ichneumonidae | Kent 1996[17] |
| *Plutella xylostella* | Yes | *Diadegma tibialis* | Ichneumonidae | Gauld 1984[1] |
| *Plutella xylostella* | Yes | *Diadromus collaris* | Ichneumonidae | Kent 1996[17], Gauld 1984[1], Hamilton 1979[15] |
| *Plutella xylostella* | Yes | *Eriborus* sp. | Ichneumonidae | Kent 1996[17], Gauld 1984[1] |
| *Plutella xylostella* | Yes | *Eriborus* sp. | Ichneumonidae | Gauld 1984[1] |
| *Plutella xylostella* | Yes | *Lienella* sp. | Ichneumonidae | Kent 1996[17], Gauld 1984[1] |
| *Plutella xylostella* | Yes | *Megadicylus dubius* | Pteromalidae | Kent 1996[17] |
| *Plutella xylostella* | Yes | *Paraphylax corvax* | Ichneumonidae | Kent 1996[17] |
| *Plutella xylostella* | Yes | *Paraphylax sp 23.* | Ichneumonidae | Gauld 1984[1] |
| *Plutella xylostella* | Yes | *Pediobius* sp. | Eulophidae | Kent 1996[17] |
| *Plutella xylostella* | Yes | *Stichtopisthus* sp. | Ichneumonidae | Kent 1996[17] |
| *Plutella xylostella* | Yes | *Trichomalopsis* sp. | Pteromalidae | Kent 1996[17] |
| *Spodoptera litura* | Yes | *Meteorus pulchricornis* | Braconidae | Suzuki & Tanaka (2007)[18] |
| *Zermizinga sinuata* | Yes |  |  |  |
| *Zizina labradus* | Yes | *Apanteles* sp. | Braconidae | Johnson *et al.*  2000[19] |
| *Zizina labradus* | Yes | *Aplomya* sp. | Tachinidae | Cantrell 1986[2] |
| *Zizina labradus* | Yes | *Carcelia (Senometopia)* sp. | Tachinidae | Cantrell 1986[2] |
| *Zizina labradus* | Yes | *Paradrino laevicula* | Tachinidae | Cantrell 1986[2] |

^1^http://www.nzffa.org.nz/farm-forestry-model/the-essentials/forest-health-pests-and-diseases/Pests/Strepsicrates-macropetana

^2^This is not a complete citation list, we focussed on obtaining at least one citation for the interaction.

**REFERENCES**

1. Gauld ID (1984) An introduction to the Ichneumonidae of Australia. London: British Museum (Natural History).

2. Cantrell BK (1986) An updated host catalog for the Australian Tachinidae (Diptera). Journal of the Australian Entomological Society 25: 255-265.

3. Gamez-Virues S, Gurr GM, Raman A, La Salle J, Nicol H (2009) Effects of flowering groundcover vegetation on diversity and activity of wasps in a farm shelterbelt in temperate Australia. Biocontrol 54: 211-218.

4. James DG, Stevens MM (1992) *Stenomesius japonicas* (Ashmead) (Hymenoptera, Eulophidae), a parasitoid of the introduced biological control agent *Dialectica scalariella* (Zeller) (Lepidoptera, Gracillariidae). Journal of the Australian Entomological Society 31: 233-234.

5. Berndt LA, Allen GR (2010) Biology and pest status of Uraba lugens Walker (Lepidoptera: Nolidae) in Australia and New Zealand. Australian Journal of Entomology 49: 268-277.

6. Mansfield S, Kriticos DJ, Potter KJB, Watson MC (2005) Parasitism of gum leaf skeletoniser (Uraba lugens) in New Zealand. New Zealand Plant Protection 58: 191-196.

7. Austin AD, Dangerfield PC (1993) Systematics of Australian and New Guinean Microplitis Foerster and Snellenius Westwood (Hymenoptera: Braconidae: Microgastrinae), with a review of their biology and host relationships. Invertebrate Taxonomy 7: 1097-1166.

8. Common IFB (1990) Moths of Australia: Melbourne University Press. i p.

9. Paull C, Austin AD (2006) The hymenopteran parasitoids of light brown apple moth, Epiphyas postvittana (Walker) (Lepidoptera : Tortricidae) in Australia. Australian Journal of Entomology 45: 142-156.

10. Walker PW (2011) Biology and development of Chaetophthalmus dorsalis (Malloch) (Diptera: Tachinidae) parasitising Helicoverpa armigera (Hübner) and H. punctigera Wallengren (Lepidoptera: Noctuidae) larvae in the laboratory. Australian Journal of Entomology 50: 309-318.

11. Johns CV, Whitehouse MEA (2004) Mass rearing of two larval parasitoids of Helicoverpa spp. (Lepidoptera : Noctuidae): Netelia producta (Brulle) and Heteropelma scaposum (Morley) (Hymenoptera : Ichneumonidae) for field release. Australian Journal of Entomology 43: 83-87.

12. Bishop AL, McKenzie HJ (1991) Key mortality factors of Merophyas divulsana (Walker) (Lepidoptera: Tortricidae) larvae in the Hunter Valley. General and Applied Entomology 23: 59-64.

13. Hardwick S (2006) Parasitoids and hyperparasitoids of Mythimna convecta (Walker) (Lepidoptera : Noctuidae) larvae infesting late-maturing maize in southern New South Wales. Australian Journal of Entomology 45: 96-100.

14. Jones RE (1987) Ants, parasitoids, and the cabbage butterfly *Pieris rapae*. Journal of Animal Ecology 56: 739-749.

15. Hamilton JT (1979) Seasonal abundance of Pieris rapae (L.), Plutella xylostella (L.) and their diseases and parasites. General and Applied Entomology 11: 59-66.

16. Keller MA (1990) Responses of the parasitoid *Cotesia rubecula* to its host *Pieris rapae* in a flight tunnel. Entomologia Experimentalis Et Applicata 57: 243-249.

17. Kent JK (1996) The taxonomy of the hymenopteran parasitoid complex associated with diamondback moth, *Plutella xylostella* (L.) in Australia. Adelaide: The University of Adelaide. 102 p.

18. Suzuki M, Tanaka T (2007) Development of Meteorus pulchricornis and regulation of its noctuid host, Pseudaletia separata. Journal of Insect Physiology 53: 1072-1078.

19. Johnson M-L, Pearce S, Wade M, Davies A, Silberbauer L, et al. (2000) Review of Beneficials in Cotton Farming Systems. Narrabri: Cotton Research and Development Corportation.
